# Interest to better investiguate self-recognition and self/other distinction impairments in neurodevelopmental and psychiatric disorders using the double Mirror ALTER EGO paradigm

**DOI:** 10.1192/j.eurpsy.2025.1840

**Published:** 2025-08-26

**Authors:** N. Lavenne - Collot, E. Maubant, M. Tersiguel, S. Deroulez, G. Bronsard, M. Wehrmann, M. Botbol, A. Berthoz, L. Vaivre-Douret

**Affiliations:** 1 University of Paris Cité, Faculty of Societies and Humanities, Institute of Psychology, Doctoral School 261-3CH; 2 INSERM Unit 1018-CESP, Faculty of Medicine, University of Paris-Sarclay , UVSQ, Villejuif, Research Team “Neurodevelopmental and learning disabilities, Necker-Enfants Malades hospital, Paris; 3 INSERM Unit 1101 Laboratory of medical information processing (LaTim), University of Western Brittany; 4 Department of Child and Adolescent Psychiatry, University hospital center of Brest; 5 Faculty of Medicine, University of Western Brittany; 6 Associated Team research 7479 (SPURBO), Faculty of Medicine, University of Western Brittany, Brest; 7 Collège de France, Laboratory of Physiology of Perception and Action, Unit 7152 CNRS, Paris, France; 8 Bauhaus Universität, Weimar, Germany; 9 Collège de France, Interdisciplinary Center of Biology; 10 University of Paris Cité, Faculty of Health, Department of Medicine Paris Descartes; 11 Institut Universitaire de France (IUF), Chair of Neurodevelopmental Clinical Phenotyping; 12 AP-HP, Necker Enfants Malades University hospital; 13 IMAGINE Institut, Department of Endocrinology, Necker-Enfants Malades hospital, Paris, France

## Abstract

**Introduction:**

In recent years, the concept of Self/Other distinction (SOD),the ability to differentiate one’s own body, actions and mental states from those of others,has recieved increasing interest. Studies that have explored SOD in psychiatric or neurodevelopmental disorders generally used static images or movies that progressively morph from one’s own face to another’s. However, these paradigms may be insufficient for SOD assessment given the centrality of embodiment in the pathophysiology of these disorders.The new Alter Ego double mirror paradigm was developped to specifically explore SOD under greater ecological conditions.This innovative device allows the progressive morphing of one’s own face to that of another between two subjects physically facing each other on either side of the device.

**Objectives:**

Two pilot studies were conducted to investigate self-recognition and self/other distinction respectively in adolescents with Anorexia nervosa (AN) and Autism Spectrum Disorder (ASD) compared with matched healthy controls through a self/other face identification task using the “Alter Ego” TM double mirror system.

**Methods:**

Participants had to watch a double mirror in which their own face was gradually morphed into the face of an unfamiliar other (self-to-other) or vice versa (other-to-self ), requiring them to indicate at which point they judged the morph to look more like their own face than the other’s face.Two judgment criteria were used: 1) M1: Threshold at which the subject starts recognizing his own face during the other-to-self morphing sequence 2) M2: Threshold at which the subject starts recognizing the other’s face during the self-to-other morphing sequence. For participants with AN, in a second part, SOD was reassessed during five different sensorimotor tasks aimed at increasing body self-consciousness.

**Results:**

Compared to controls, participants with ASD and AN showed an earlier self recognition in the other-to-self direction and a delayed other recognition in the self-to-other direction. Moreover, contrasting with controls, in ASD and AN participants, the critical threshold for switching between self and other varied with the direction of morphing. Finally, when participants with AN were seated with a backrest and footrest reinforcing the median axis of their body, the self-recognition threshold (M1) increased significantly approaching that of controls.

**Conclusions:**

The preliminary results of these studies uncovered novel findings showing first behavioral evidence of impaired self/other distinction in individuals with ASD and AN through an embodied face-recognition paradigm. These results confirm the interest of the Alter Ego double mirror paradigm for the study of alterations in self-consciousness and Self/Other Distinction as a transosographic dimension common to various neurodevelopmental or psychiatric disorders.

**Disclosure of Interest:**

None Declared

